# Around the Hospitals

**Published:** 1894-09-15

**Authors:** 


### Around the Hospitals.

[Notice to the Managers of Institutions.—Special space is now reserved for the insertion of notices of hospital meetings and festivals. The Editor requests that all notices may reach The Hospital Office, 428, Strand, at least one week before the date of each meeting.]

**The Home and Hospital for Jewish Incurables.**—Such homes are of the utmost blessing to the poor, whom they remove from being a burden to those with whom they live, and who are little able to care for them. The Jewish home contains 24 inmates. There is space for a larger number, but finances do not allow of their reception. The Home has suffered from disputes which have arisen amongst the community, but that impediment is now removed, and the good work of the institution may be expected to continue unimpeded. A novel mode of augmenting the finances of the home, and enlarging its sphere of interest, is the formation of what is called a "Ten Club." It appears that a large number of shilling tickets are sold, and when a sufficient sum has been collected a ballot is held, the winner becoming a life-governor of the institution. Six governors have so far been created in this manner. It is the wish of the committee to admit children as inmates, but this is not possible in the present financial condition of the home. The expenditure for the year was £973, showing a reduction on that of the previous year, in spite of an increase in the number of patients.

**City of London Hospital for Diseases of the Chest.**—Since last year the north tower and sanitary improvements connected with it have been completed, and the hospital arrangements rendered far more satisfactory. A further improvement will be the addition of the proposed outh tower, but this cannot at present be proceeded with as funds will not permit. To clear off expenses connected with the building of the north tower, the small reserve fund of the institution had to be encroached upon to the extent of over £2,000, and further, a loan of £1,500 was incurred. The income of the hospital shows a diminution, in spite of the increase in the number of both in and out patients, and that the efficiency of the hospital continues satisfactory. During the year the institution lost three valued friends—Mr. Odling, the treasurer, and Sir Andrew Clark, honorary consulting physician, by death, and Dr. Omerod, physician since 1879, by retirement. Early in the year the hospital ran the danger of being demolished by fire. Owing to the promptitude of the staff the fire was quickly got under, and no fatality occurred. The number of in-patients received amounted to 1,292, and in the out-patients' department 16,976 attended. The total income amounted to £8,892. Of this only £2,411 was received from annual subscriptions, whilst legacies brought in £3,807. The ordinary expenditure was £11,270, and the extraordinary equalled £1,429. The hospital is situated in a part of London little known to dwellers in richer neighbourhoods, and its needs are therefore liable to be forgotten. Existing where it does, however, it is of the greatest use to the poor, by whom it is increasingly appreciated, and for whose sake it is to be hoped it will receive the large support it deserves.

**St. George's Hospital.**—We are again reminded that St. George's Hospital is not a rich institution, a fact which the public apparently do not yet grasp, seeing that it is situated in so rich a neighbourhood. St. George's requires a large income, amounting to £30,000, to maintain it in efficiency. Of this sum last year little over £6,000 was secured by subscriptions. Much larger contributions and donations are required to meet the wants of the hospital, for the estimated £30,000 applies only to ordinary expenditure, and during the past year over £12,000 was required beyond this sum for structural and other alterations and improvements. To secure this sum £12,000 of stock were sold, a serious diminution of invested funds. A large number of the in-patients and all the out-patients are received without letters of recommendation. During the past year the work of the hospital was very heavy in both departments. In spite of the closing of the wards for a time, an increase in the number of in and out patients is to be noticed, and the accommodation proved insufficient on several occasions. Several changes in the medical department have taken place. Mr. Brudenell-Carter on his retirement, after valuable service for twenty-three years, has been appointed consulting ophthalmic surgeon. Mr. William Fuller, the visiting apothecary, who benefited the hospital by his assistance for twenty-seven years, was removed by death. One assistant physician and one assistant surgeon have been added to the visiting staff. During the year in-patients numbered 4,700 and the out-patients 34,860. The total ordinary income was £25,857 and the total ordinary expenditure £30,788.

